# Time-Constrained Adversarial Defense in IoT Edge Devices through Kernel Tensor Decomposition and Multi-DNN Scheduling

**DOI:** 10.3390/s22155896

**Published:** 2022-08-07

**Authors:** Myungsun Kim, Sanghyun Joo

**Affiliations:** 1 Department of Applied Artificial Intelligence, Hansung University, Seoul 02876, Korea; 2 Department of IT Convergence Engineering, Hansung University, Seoul 02876, Korea

**Keywords:** IoT edge device, embedded GPU, approximate computing, tucker decomposition, GPU scheduling framework, adversarial defense

## Abstract

The development of deep learning technology has resulted in great contributions in many artificial intelligence services, but adversarial attack techniques on deep learning models are also becoming more diverse and sophisticated. IoT edge devices take cloud-independent on-device DNN (deep neural network) processing technology to exhibit a fast response time. However, if the computational complexity of the denoizer for adversarial noises is high, or if a single embedded GPU is shared by multiple DNN models, adversarial defense at the on-device level is bound to represent a long latency. To solve this problem, eDenoizer is proposed in this paper. First, it applies Tucker decomposition to reduce the computational amount required for convolutional kernel tensors in the denoizer. Second, eDenoizer effectively orchestrates both the denoizer and the model defended by the denoizer simultaneously. In addition, the priority of the CPU side can be projected onto the GPU which is completely priority-agnostic, so that the delay can be minimized when the denoizer and the defense target model are assigned a high priority. As a result of confirming through extensive experiments, the reduction of classification accuracy was very marginal, up to 1.78%, and the inference speed accompanied by adversarial defense was improved up to 51.72%.

## 1. Introduction

**Figure 1:**
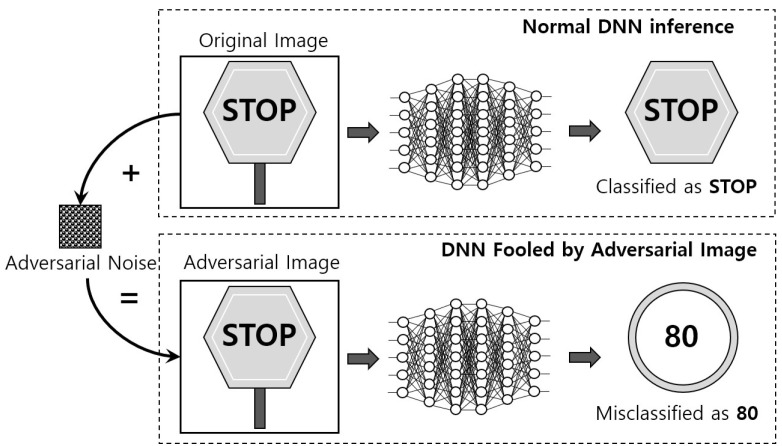
Example of an adversarial attack to an autonomous vehicle.

Artificial intelligence (AI) services triggered by deep learning technologies are making our daily lives more convenient. AI applications based on deep learning models have been on the surface in various critical fields, e.g., in medical image analysis [1], construction [2], self-driving cars [3], and metaverse-based education [4]. Deep learning technology has its good sides, but it is susceptible to adversarial attacks. Adding a very small amount of perturbation that cannot be identified with the human eyes to the original image becomes an adversarial example, which can make a fool of the deep learning model [5,6,7]. Thus, lack of robust and properly implemented defense mechanisms leaves deep learning applications vulnerable to adversarial attacks. Furthermore, in security-sensitive applications such as autonomous driving and invasive surgery, this malicious attack can lead to dreadful consequences. An example of an adversarial attack to an autonomous vehicle is shown in Figure 1. An attacker can intercept transmission from the camera and disturb the image by adding adversarial noises (perturbation) before being delivered to the DNN-based object detection system. Alternatively, by manipulating road signs, devices that perform DNNs, although completely understandable to humans, can be confused and completely misclassified.

To defend against such attacks, a method of eliminating malicious noise before the target inference starts is naturally required. In general, just as deep learning models require very high model-complexity to maintain high recognition accuracy, a denoizer also requires a high degree of computational complexity to maintain high restoration accuracy through denoizing [8]. In this environment, the target inference time is the sum of the denoizing time and the pure inference time, and therefore the time required to perform denoizing should be minimized.

In an effort to tackle network latency and security issues, IoT edge devices usually perform operations which are required for deep learning models on their own in a cloud server-independent form. An IoT edge platform such as the Jetson AGX Xavier platform [9] usually consists of an integrated (embedded) GPU and several CPUs, and due to its excellent parallel processing capability in MAC (multiply–accumulate) operations, most DNN (deep neural network) operations are performed on the GPU. Presently, several deep learning models are being performed simultaneously to meet improved AI service quality and various needs. These conditions, however, can make the GPU a performance bottleneck.

When a denoizer is required to defend against malicious attacks and multiple deep learning models are performed together, a critically important denoizer should be treated as a top priority. However, since the GPU is an inherently non-preemptive device and executes DNN kernels with a FIFO order, it is difficult to guarantee the preferential execution of DNN operations necessary for the denoizer.

To tackle those issues mentioned above, in this study, we propose eDenoizer. It is an embedded denoizer especially designed for embedded systems such as IoT edge devices. First, our study adopts [8] as a baseline denoizer. Then, an approximate technique is applied to reduce the model-complexity of the baseline denoizer. This overcomes the limited computational capability of the embedded environment. Second, we propose a GPU scheduling framework that reflects the priority specified by the CPU-side OS (operating system) so that the critical task such as the denoizer can be preferentially handled by the GPU. Third, we propose a mechanism that can maximize the parallel processing capability of the integrated GPU in an environment where several deep learning models are performed together. Through this, the denoizer and the target inference using the output of the denoizer can be accelerated simultaneously.

The rest of this paper is structured as follows. Section 2 provides the technical background of this study to help in understanding eDenoizer. Section 3 states formally our problem to solve. Section 4 details the solution architecture and then technically treats the proposed solution. Section 5 reports on the experiments to evaluate eDenoizer. Finally, the conclusions are given in Section 6.

## 2. Background

### 2.1. Adversarial Attack and Defense

Adversarial examples [10] can be inputs, including image data, maliciously crafted to lead to a wrong classification result. They usually have quite a small difference from the original data, and the difference is made up of some amounts of perturbation. Given an input data *x*, a deep learning model is defined as D:x→y, and J(x,y) specifies the loss function applied for the training step of model *D*. Here we denote $x^{*}$ as the adversarial example developed from the clean image *x* [8]. Using adversarially manipulated $x^{*}$, a certain datapoint *x* can be forced to be classified into a wrong class instead of *y*, and we call this an adversarial attack.

An attack algorithm called the Fast Gradient Sign Method (FGSM) was proposed, and an adversarial example $x^{*}$ is obtained from Equation (1) [8,11].

(1) $$x^{*}=x+\epsilon \cdot sign\left({▽}_{x}J\left(x,y\right)\right)$$

where ϵ denotes the degree of adversarial perturbation added on. To derive a higher misclassification rate, an iterative FGSM was proposed by [12] which repeats FGSM several times. In [8], this iterative FGSM is referred to as IFGSM, and if FGSM is repeated by *n* steps, it is expressed as IFGSMn.

Depending on how well the adversary knows the target model, attacks can be classified into two categories: white-box attacks and black-box ones. The former is an attack when the attacker knows all the information in the target deep learning model, e.g., the neural network architecture, parameters, and gradients of the model. Thus, the adversarial example generator contains the target model itself. Instead, as for a black-box attack, the attacker has no knowledge of the target deep learning model. Attacks are accomplished only relying on the in/out pattern of the target model and examples are generated on other deep learning models. Therefore, black-box attacks are more difficult than white-box attacks [8,13,14].

### 2.2. Denoiser

To firmly defend against such adversarial attacks, HGD (high-level representation guided denoizer) [8], a denoizing network architecture in the circumstances under image classification using the ImageNet dataset [15] has been proposed. The crux of HGD is DUNET, which is a combination of denoizing autoencoder (DAE) [16] and U-net [17]. The key functional block that performs denoizing inside HGD is DUNET. To fool a deep learning model, attackers add adversarial noises to clean images. In this respect, the basic idea of HGD is to denoize maliciously crafted examples before they are forwarded to the target inference engine (i.e., defense target model). HGD can effectively remove error amplification effect which means even a small amount of residual perturbations can be augmented in the top layers [8]. So HGD can work like that, the loss function of HGD takes $L_{1}$ norm of the difference between the outputs of the last layers of the target deep learning model generated from both clean and adversarial examples [8]. Moreover, HGD is highly defensive against both white-box and black-box attacks and is applicable to various deep learning models.

**Figure 2:**
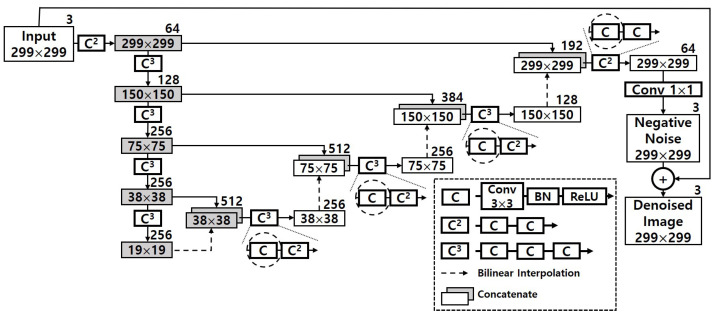
Denoizing sequence of DUNET in HGD [8].

Figure 2 pictorially shows the detail of the denoizing sequence in HGD. The numbers inside each rectangle stand for the sizes of feature maps and images, i.e., width × height, and the number outside the rectangle denotes the number of channels. *C* means a series of layers, which consists of a 3 × 3 convolution, a BN (batch normalization) layer and a ReLU (rectified linear unit). $C^{r}$ is defined as *r* consecutive *C*, and Conv k×k means a k×k convolutional layer. The final output of DUNET is the denoized image which has the same resolution with the input image. To obtain the denoized image, DUNET first makes the negative noise and then add this to the input image [8]. The circled sections drawn in dashed lines in the figure represent the first ones of two or three consecutive convolutional layers. As can be seen indirectly from the figure, the number of their output channels is 256, 256, 128 and 64, respectively, indicating a relatively large computational scale. These fours dashed circles represent the portion that accounts for about 40.37% of the total operation. Therefore, in order to realize HGD in resource-limited embedded systems, it is necessary to be able to efficiently reduce the computation volume of the corresponding part while minimizing the accuracy loss.

### 2.3. Tucker Decomposition on Convolution Kernel Tensors

Tucker decomposition has provided an effective mean to reduce the computational volume of deep learning models by restructuring the convolutional hidden layers which occupy the majority of the computation [18]. In a deep learning model, a convolutional layer usually adopts a 4-way kernel tensor of size $k_{w}\times k_{h}\times c_{i}\times c_{o}$ where $k_{w}/k_{h}$ is the kernel width/height and $c_{i}/c_{o}$ means the number of input/output channel. By applying Tucker decomposition, we can obtain a core tensor *g* size of $R_{1}\times R_{2}\times R_{3}\times R_{4}$ and four factor matrixes ($A_{1}$∼$A_{4}$) sizes of $k_{w}\times R_{1}$, $k_{h}\times R_{2}$, $c_{i}\times R_{3}$ and $c_{o}\times R_{4}$, respectively, ref. [19]. Here, $R_{1}$∼$R_{4}$ mean the rank induced from each way of the kernel.

**Figure 3:**
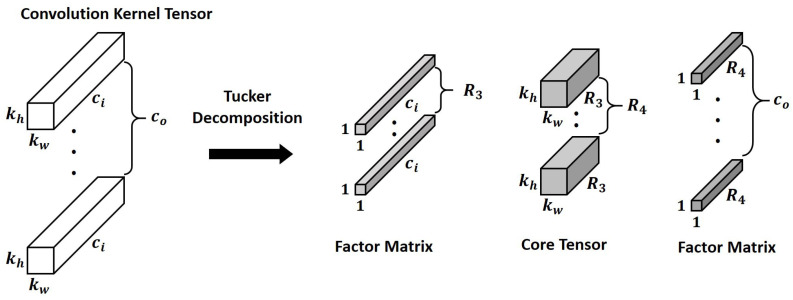
Tucker decomposition on a convolution kernel tensor [19].

When we apply Tucker decomposition on convolution kernel tensors, we do not have to apply it to every way of the kernel tensor. For instance, $k_{w}$ and $k_{h}$ are usually the same and the spatial dimension of them are typically small by 3 or 5. Thus, we only takes into account the ways of $c_{i}$ and $c_{o}$. Subsequently, the decomposition result is that the size of the core tensor *g* is $k_{w}\times k_{h}\times R_{3}\times R_{4}$, and two factor matrixes sizes of $c_{i}\times R_{3}$ and $c_{o}\times R_{4}$. As a result, a 4-way $k_{w}\times k_{h}\times c_{i}\times c_{o}$ sized kernel tensor is decomposed into three 4-way tensors such that $1\times 1\times c_{i}\times R_{3}$, $k_{w}\times k_{h}\times R_{3}\times R_{4}$ and $1\times 1\times c_{o}\times R_{4}$, and Figure 3 details the decomposition result.

In terms of complexity analysis according to the number of parameters, originally $k_{w}\times k_{h}\times c_{i}\times c_{o}$ parameters are required. With Tucker decomposition, the number of parameters of the kernel tensor is reduced by $c_{i}\times R_{3}+k_{w}\times k_{h}\times R_{3}\times R_{4}+c_{o}\times R_{4}$ where generally $c_{i}$ is sufficiently larger than $R_{3}$ and also $c_{o}$ is much larger than $R_{4}$.

## 3. Problem Formulation

### 3.1. Problem Description

To guarantee a correct classification result, typically denoizing procedure is performed prior to the inference step. So the execution time of DUNET in HGD is critical in terms of the system-wide inference performance. As Figure 2 shows, in DUNET, several large convolutional layers are stacked, which means that DUNET itself is computationally expensive. To quantitatively identify this, we measured the time DUNET took for processing a single input image as well as the times of three other DNN models. The three DNN models were Inception-V3 [20], ResNet-152 [21] and VGG-16 [22], and they can be the defense target models. We ran DUNET and the three defense target models on Jetson AGX Xavier [9] and Jetson TX2 [23] platforms, which are representative COTS (commercial Off-the-shelf) IoT edge platforms. Table 1 describes the result.

Once an adversarial example is entered, denoizing is performed first via DUNET, and then the output of DUNET is entered into the defense target model. Thus, the overall inference time for one input image should add up to the time it takes to process one image in DUNET and the time it takes in the defense target model. If one input image is inferred via Inception-V3 on the Jetson AGX Xavier platform, approximately twice as much time should be spent on DUNET first. The output of the DUNET is then input to Inception-V3, which performs pure inference. As a result, the total inference time is about 247 ms. In Jetson TX2, it can be seen that DUNET consumes more than twice as much time as Inception-V3, resulting in an overall inference time of about 350.3 ms.

The second problem is found in terms of the use of the deep learning computing unit inside IoT edge devices. The Jetson AGX Xavier platform [9] is an representative IoT edge device installed in unmanned vehicles distinctly designed for deep learning applications, being equipped with 8 ARM cores and an embedded GPU core. Due to the support such as runtime and libraries [24,25], application-level programmers can easily entrust accelerated processing of deep learning applications to the embedded GPU in the Jetson AGX Xavier platform. That GPU basically supports hardware-level multi-threading, as other discrete GPUs used in server computing systems. It also supports the CUDA stream technology [26], enabling simultaneous processing of deep learning kernels commissioned by multiple processes within the limits permitted by registers and streaming multiprocessors (SMs).

Despite this excellent parallel processing capability, when multiple deep learning kernel-launch happens simultaneously, performance bottleneck on the single embedded GPU is inevitable [27]. Furthermore, the GPU by nature is non-preemptive, thus, even if a high priority is given to DUNET performing denoizing, DUNET execution is delayed if another model arrives first at the execution engine (EE) queue of the GPU. Generally, deep learning models created from frameworks such as PyTorch [28] and TensorFlow [29] are executed in a way of batch processing method [30], i.e., the execution unit is an entire model. In this case, head-of-line blocking problem inevitably happens [31].

### 3.2. Problem Statement

**Figure 4:**
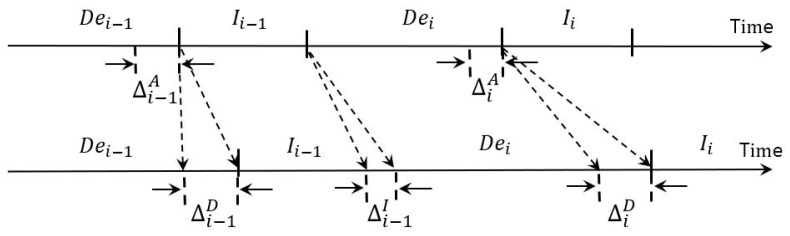
Illustration of the denoizing phase of DUNET and the inference phase of the defense target model for continuous image inputs.

To avoid attacks by maliciously crafted examples, DUNET renders every incoming image undergo two serialized steps: denoizing and inference with the denoized image, and Figure 4 shows the frame sequence of incoming images where $De_{i}$ and $I_{i}$ mean the denoizing and the inference steps of the *i*th image. The upper time line depicts when DUNET does not experience any delay while the bottom one represents a more realistic execution environment, when other co-runners are concurrently executed.

In the figure, $\Delta_{i}^{D}$ means the amount of delay for the *i*th denoizing step, and $\Delta_{i}^{I}$ is for the *i*th inference time of the defense target model, respectively. The cause of these delays are mostly that DUNET and target deep learning models are not delivered preferentially to the GPU EE queue. Meanwhile, $\Delta_{i}^{A}$, represents the *i*th amount of time that can be reduced when using lightweight approximated DUNET which has the shrunk computational complexity compared to the original DUNET. By adding the times taken for the denoizing and inference steps of the defense target model, we can represent the total time for *i*th target inference as $De_{i}-\Delta_{i}^{A}+\Delta_{i}^{D}+I_{i}+\Delta_{i}^{I}$. We define the average target inference time of a DNN model with *F* incoming frames of images as follows.

(2) $$Inf_{avg}\left(F\right)=\left\{\frac{\sum_{i=1}^{F}\left(De_{i}-\Delta_{i}^{A}+\Delta_{i}^{D}+I_{i}+\Delta_{i}^{I}\right)}{F}\right\}$$

Obviously, our problem to solve is minimizing $Inf_{avg}\left(F\right)$.

One more noticeable fact is that $De_{i}$ and $I_{i-1}$ are not data dependent. That is, these two steps can be overlapped. In addition, if $\Delta_{i}^{A}$ in Figure 4 is large enough by applying the approximation method, but within the range that the accuracy drop is marginal, each $De_{i}$ of DUNET can be hidden by $I_{i-1}$. Consequently, the total execution time of DUNET can be only in time of $\sum I_{i}$.

## 4. eDenoizer

This section gives an explanation of the proposed approach, called eDenoizer. We first overview the overall operational sequence of it and then elaborate on the technical details.

### 4.1. Solution Overview

**Figure 5:**
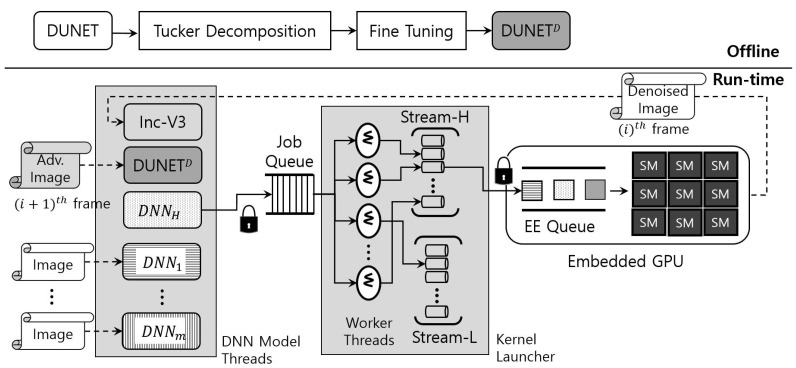
Operational workflow of eDenoizer.

Figure 5 details the operational workflow of eDenoizer. A rough look at the proposed approach shows that it consists of the offline procedure and the run-time one. In offline, DUNET is approximated through Tucker decomposition. Next, after performing training through the post hoc fine-tuning step, the accuracy loss is minimized and DUNET^D^ is obtained. In run-time mode, multiple deep learning models and DUNET are executed over a scheduling framework, which consists largely of a set of DNN model threads, a job queue, and a kernel launcher. DNN model threads are composed of deep learning models, each of which is implemented as a thread and is a target scheduled to the CPUs over the underlying OS (operating system). Each model thread can asynchronously request its own DNN operation to the job queue. The kernel launcher extracts the requested operation from the job queue through the worker threads that exist inside it and enqueues it to the EE queue of the GPU.

The computational output from the GPU is the result of the requested operation of a DNN model, including DUNET, which is mainly feature map data (mostly an intermediate result). This value is stored in the system memory, fed back to the set of DNN model threads, and then input to the corresponding model. At this time, if the result value is a denoize image, which is the final output of DUNET, it is not given as a DUNET input again, but as an input to the DNN model (e.g., Inception-V3 [20] in Figure 5) that performs target inference.

As mentioned earlier in Section 3, if DUNET does not finish its denoizing step for the (i)th image, the (i)th target inference step cannot occur. In addition, the (i+1)th DUNET processing step and the (i)th target inference step have no data dependency. Thus, as shown in Figure 5, both can be handled asynchronously and simultaneously in the scheduling framework. It is noteworthy that, and as described above, since the processing of DUNET was shortened with Tucker decomposition, the total target inference time is only bounded by the target inference time (i.e., (i+1)th DUNET step is hidden by (i)th target inference).

### 4.2. Scaling down the Computational Scale of DUNET

**Figure 6:**
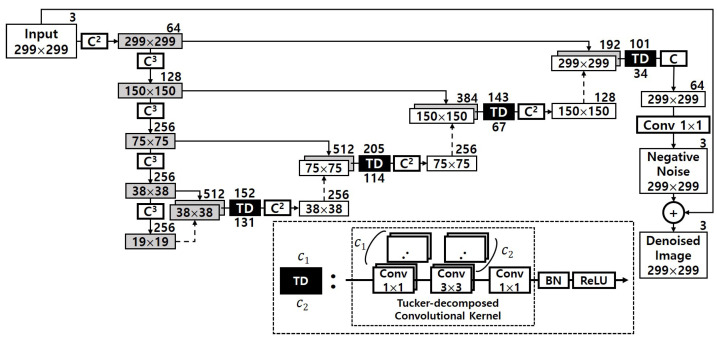
Approximate DUNET via Tucker decomposition.

As detailed in Figure 4, the first hurdle we want to overcome is to maximize $\Delta_{i}^{A}$. To do so, we shrink the computational complexity of the original DUNET by applying an approximate computing method using Tucker decomposition, and Figure 6 describes the result of it. Since the four circles drawn with dashed lines in Figure 2 take over more than 40% out of the total amount of computations, those 3 × 3 convolutional layers need to be changed to have a structurally small computational quantity. Each of the four convolutional layers in Figure 2 is changed into two 1 × 1 convolutional layers and one smaller 3 × 3 convolutional layer, and the four black boxes with ‘TD’ in white letters in Figure 6 imply those Tucker-decomposed convolution kernel tensors.

To take advantage of dimension reduction, the two 1 × 1 convolutional layers (the two factor matrixes in Figure 3) are given to the both sides of the 3 × 3 convolutional layer (the core tensor), which is the same as the bottleneck building block in ResNet-152 [21]. The numbers above each black box represents the number of the output channel in each first 1 × 1 Tucker-decomposed convolution kernel tensor, and the numbers below represents the number of the output channel of the 3 × 3 core tensor which is immediately adjacent to the first 1 × 1 kernel tensor. The total computation volume is reduced by changing the number of large output channels pointed out in Figure 2 to $c_{1}$ and $c_{2}$.

**Figure 7:**
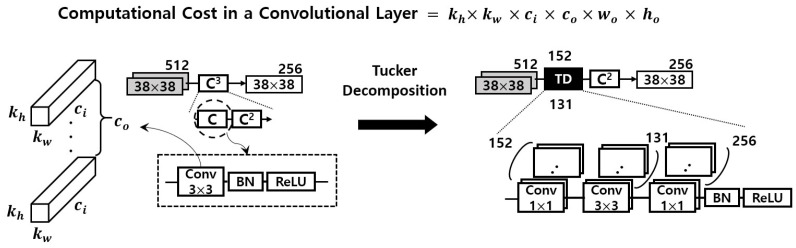
Tucker decomposition result of the first of the convolutional layers requiring computational reduction inside DUNET.

For instance, the left first circle drawn with dashed line in Figure 2 has one 3 × 3 kernel tensor with 256 output channels. As shown in Figure 3, it is decomposed into three small convolution tensors, and the number of the output channel of each tensor is 152, 131, and 256, respectively. To explain this more clearly and quantitatively, we analyze the reduced computation amount, and that part is shown again in Figure 7. The computational cost of one convolutional layer is obtained by the formula shown in the figure. It can be seen that the convolutional layer, which originally had an arithmetic amount of $k_{h}\times k_{w}\times c_{i}\times c_{o}\times w_{o}\times h_{o}$ = 3 × 3 × 512 × 256 × 38 × 38 = 1,703,411,712, is reduced to 1 × 1 × 512 × 152 × 38 × 38 + 3 × 3 × 152 × 131 × 38 × 38 + 1 × 1 × 131 × 256 × 38 × 38 = 419,580,192. This represents a decrease of about 75.4% considering only the corresponding convolutional layer. When the same is applied to the remaining three convolutional layers, the overall system-wide computational reduction is confirmed to be about 25.41%, and DUNET^D^ in Figure 5 denotes the final output of approximate DUNET.

### 4.3. Scheduling Framework for Multiple Deep Learning Models

As described in Figure 4, our second objective is to keep $\Delta_{i}^{D}$ and $\Delta_{i}^{I}$ minimized while DUNET, the defense target model performing inference using the result of DUNET and various other deep learning models, are performed simultaneously with each other.

Once the system is started, the deep learning models to be performed are loaded from the system memory to the GPU memory. Please note that IoT edge devices use embedded GPUs. Furthermore, they do not have a separate dedicated memory and shares the system memory referenced by the CPU. Therefore, when deep learning models are loaded into GPU memory, data copy does not occur, and instead only memory location information (i.e., pointer) is delivered from the memory set on the CPU side [27].

#### 4.3.1. Scheduling Unit

To discuss the scheduling unit of DNN model threads, if an entire model is pushed through to the job queue, a head-of-line blocking problem arises, which leads to a scheduling latency for high priority deep learning models, whereas, if too small a unit is scheduled, it can trigger OS interventions too frequently, causing overhead. Thus, the scheduling unit of a single DNN model thread to be delivered to the job queue is a critical factor for system-wide performance. For example, convolutions and activation functions differ greatly in their computational scale. There is a big difference in terms of system-wide performance between two convolutions as one scheduling unit and each of the convolution and activation functions as a distinct scheduling unit. Moreover, the structure of each deep learning model and the computational scale required for each DNN operation are different. Therefore, it is undesirable to apply the scheduling unit by applying a uniform rule to all deep learning models.

Therefore, in this study, the optimal scheduling unit for each deep learning model including DUNET is first determined offline. A job in Figure 5 means this scheduling unit. For example, in some models, one convolution can be a job, and in others, convolution, BN (batch normalization), and an activation function such as ReLU (rectified linear unit) can be combined to form a job.

#### 4.3.2. Scheduling Algorithm

Algorithm 1 shows the details of the scheduling framework through a clear-cut and unambiguous pseudo-code. Each of the individual DNN model thread in Figure 5 is largely implemented in two functional blocks, and **construct_layers**( ) and **execute_dn**( ) in Algorithm 1 describe their behavior. First, considering the different features of each deep learning model, $layers_{id}\left[l\right]$ is filled with $L_{id}^{l}$ by referring to the configuration information on each model defined offline where *l* means the layer index and id does the DNN identification number (lines 2∼4). $L_{id}^{l}$ may consist of several DNN operations, and for example, a 3 × 3 convolution, a BN layer and ReLU can comprise $L_{id}^{l}$.
**Algorithm 1** Multi-DNN Scheduling Framework1:**function** construct_layers($DNN_{id}$)2:    **for** l←0 to $last_{id}$**do**            ▹$last_{id}$: last layer index of $DNN_{id}$3:          $layers_{id}\left[l\right]\leftarrow$$L_{id}^{l}$          ▹$L_{id}^{l}$: $l^{th}$ layer of $DNN_{id}$ defined offline4:    **end for**5:    **return** $layers_{id}$6:**end function** 7:**function** execute_dnn($layers_{id}$, $DNN_{id}$)8:    prev_output←inputimage9:    **for** l←0 to $last_{id}$ **do**10:        $job_{id}^{l}.layers\leftarrow$$layers_{id}\left[l\right]$11:        $job_{id}^{l}.data\leftarrow prev\text{\_}output$12:        enqueue($job_{id}^{l}$)13:        wait_signal(sig)14:        prev_output←output15:    **end for**16:**end function** 17:**function** execute_job( )18:    A:19:    **while** (job queue is empty) **do**20:        do nothing21:    **end while**22:    $job_{front}\leftarrow$ dequeue( )23:    output ← execute_kernel($job_{front}$)24:    send_signal(sig)25:    goto A:26:**end function**

Once the layer-structuring procedure mentioned above that enable a deep learning model to run in the form of a thread is completed, the model begins to run. After receiving the input image as an input, it is combined with $layers_{id}\left[l\right]$ and then a job is formed (here, *l* = 0 since the job corresponds to the first layer). Then, the job is transmitted to the job queue and waits for a signal from the kernel launcher (lines 10∼13). When a signal indicating that the launched job is completed by the GPU is received, the data output from the GPU is transmitted again as the input of the next job to be performed (lines 11, here *l* = 1). This procedure repeats until the last job with $L_{id}^{l}$ completes.

The main behavior of the kernel launcher can be seen in the function **execute_job**( ) of Algorithm 1. As can be seen from Figure 5, the kernel launcher takes out the requested job by accessing the job queue in an asynchronous manner by several worker threads (lines 19∼22). The extracted job is executed through **execute_kernel**( ) (line 23). At this time, the worker thread converts the requested job into a GPU kernel, and it is transmitted to the EE queue through one of the CUDA streams shown in Figure 5.

#### 4.3.3. Scheduling Framework Analysis

In terms of the theoretical measure of Algorithm 1, **construct_layers**( ) is not used while eDenoizer is running and is an offline process, so it is excluded from the analysis. The function **execute_job**( ) takes O(1) since it pulls out a job after looking at only the front element of the job queue. When the number of layers of the DNN model to be performed is $n=last_{id}$, **execute_dnn**( ) has a time complexity of O(n). Since any of the *n* layers cannot be omitted during the DNN model execution, the minimum time complexity that **execute_dnn**( ) may take is bound to be O(n).

In a system that operates only one deep learning model, our proposed framework is inefficient. The reason for this is the overhead that occurs in acquiring locks for the job queue and the EE queue. Conversely, the solution proposed in this study is effective in an environment in which several deep learning models, including denoizers, operate together, as shown in Figure 5. As can be seen from Figure 5, synchronization issues arise in two places. It occurs when multiple deep learning models request a job queue access and when worker threads access the EE queue through CUDA streams. These synchronization issues are problems that serialize DNN operations, which can degrade system-wide performance. In practice, however, multiple deep learning models are faster in requesting computations for consecutive input image than processing the requested deep learning operations on a single embedded GPU, so there are always several jobs in the two queues without generating any queue underflow. Therefore, synchronization primitives accessing the two queues do not affect the overall performance of the system.

#### 4.3.4. Priority-Based DNN Operations and Maximum Parallelism

The proposed framework allows deep learning models to be executed in the GPU according to the priority set by users through the CPU-side OS. To this end, the job queue is a priority queue that allows jobs thrown by DNN model threads to be arranged according to the priority of each model. Therefore, the job drawn by one of the worker threads always has the highest priority among the requested jobs. The GPU is basically non-preemptive. However, advanced architecture of NVIDIA provides preemption capability through the stream technology (only two levels: high and low) [32]. Thus, any low-stream-priority DNN operation may be preempted by a high-stream-priority DNN kernel. Moreover, each worker thread can use one of the CUDA streams separately and contemporaneously, depending on SM availability, concurrent execution of multiple jobs is possible, improving system throughput. Resultantly, $\Delta_{i}^{D}$ and $\Delta_{i}^{I}$ shown in Figure 4 can be effectively kept short.

## 5. Experiments

This section gives an explanation of experiments we have conducted to evaluate the proposed approach eDenoizer. First, we describe in detail how we implemented eDenoizer and discuss the experimental setup. We then report on various experiment results with relevant analysis.

### 5.1. Implementation

As for implementation, the GPU scheduling framework used in this study is an extension of what we used in our previous work [33]. Deep learning models in DNN model threads are generated from the PyTorch framework [28]. Since GIL (global interpreter lock) [34], which enables only one thread to handle the Python interpreter, makes it difficult to run models with pure Python in parallel, we implemented our framework using the libtorch library used in [33] to take advantage of C++. By doing so, our scheduling framework can run DNN model threads and worker threads in Figure 5 in a multithreading environment.

To the software framework used in [33], we added a routine to perform Tucker deformation offline, as shown in Figure 5. As for the layer configuration of DNN model threads, we reorganized the layer configuration inside each model thread from a uniform configuration to a different layer configuration for each model to be optimized for the execution of each model. In addition, as shown in Algorithm 1, we use a signal-based communication scheme between the worker threads and DNN model threads to make the execution of DNN operations more interactive than [33].

### 5.2. Experimental Setup

To demonstrate the efficacy of our proposed eDenoizer, we take the Jetson AGX Xavier platform as the target IoT edge device, and the detailed hardware and software specifications of the target device is described in Table 2. HGD provides three training methods. Among them, this study takes logits guided denoizer (LGD) [8]. All our experiments are performed on images from the ImageNet dataset as [8], and the same datasets applied to [8] are used for testing, training and adversarial image generation. Throughout experiments in this study, Inception-V3 is the target model of which adversarial attacks attempt to make a fool, and is also the target model that DUNET should defend.

For providing adversarial examples, we first select 30,000 samples (normal images) from the training set of ImageNet, then we distort the normal images by adding perturbations. In doing so, we use a group of attacking methods represented in Table 3. As shown in Table 3, we use three DNN models for attacked models, where attacked models mean the models being used for creating adversarial examples. As in [8], the three attacked deep learning models are Inception-V3, InceptionResnet V2 [35] and ResNet50 V2 [36], and the combination of these three models are used for ensemble adversarial training [37]. As in [8], to simplify notations, the three attacked models are represented as IncV3, IncResV2 and Res, respectively. In every training step, ϵ (perturbation level) is uniformly selected from 1∼16. The total number of training data is 240,000 including normal images. After selecting another 10,000 samples (normal images) from the training set of ImageNet, we applied the same methods used in generating training data, then the size of validation dataset is 80,000.

For constructing test dataset, as in [8], we apply two kinds of attacks as shown in Table 4: white-box attacks and block-box attacks. The two attack types are segmented by FGSM and IFGSM, respectively, to obtain different kinds of adversarial samples. In this study, if FGSM is repeated by *n* steps, it is expressed as IFGSMn as in [8].

After selecting 10,000 normal images from the ImageNet validation set, we distort them using attack methods written in Table 4. For white-box attacks, Inception-V3 is applied to generate adversarial images for test dataset, and also Inception-V3 is the target model to defend using DUNET. To contrast, for black-box attacks, Inception-V4 [35] is used for generating adversarial images, while the target model to defend is Inception-V3.

### 5.3. Classification Accuracy on Adversarial Examples

In this subsection, we verify the effectiveness of Tucker decomposition. First, we analyze how the applied approximate computing method affects the classification accuracy of DUNET, i.e., to determine how the performance of the denoizing itself has changed. Second, we compare the transferability performance of the original DUNET and the approximate DUNET. Here, transferability means to check classification accuracy by launching adversarial attacks on a deep learning model that are not used when creating training dataset. In both classification accuracy and transferability tests, ϵ (perturbation level) is set to 4.

#### 5.3.1. Classification Accuracy of Approximate DUNET on Adversarial Examples

In this experiment, we check the effect of applying three Tucker-decomposed tensors which replace the four convolutional kernel tensors of the original DUNET. Adversarial attack images are produced using White-box-test-set and Black-box-test-set shown in Table 4. For the case of adversarial images of White-box-test-set, those images are generated using Inception-V3, and the target DNN model for inference is also Inception-V3. In contrast, for the Black-box-test-set case, adversarial images are created using Inception-V4 instead of Inception-V3, and the target DNN model that performs inference under attack is Inception-V3.

Table 5 shows the classification accuracy result on adversarial images from the white-box test set and the black-box test set as well as the result with clean images. In the table, the original DUNET means the unmodified DUNET and the approximate DUNET denotes the case of applying Tucker decomposition. Each data point in the table is the average value of two attack methods in Table 4: FGSM (IncV3) and IFGSM4 (IncV3/IncResV2/Res). In White-box-test-set case, Tucker decomposition affects the classification accuracy by less than 1.78%, and for Black-box-test-set case, 0.41% performance decline is observed. These results clearly show that the denoizing performance rarely decreases even though the computational complexity of DUNET is reduced by Tucker decomposition. Furthermore, in the test result with clean images, there is almost no performance degradation (less than 0.15%).

Please note that the results in [8] in the table might be considered to be the same as the original DUNET, but they are different. The reason for this difference is that the original images of the ImageNet dataset used to create adversarial images for training and testing are randomly selected. Thus, the training and test datasets used in this study and those in [8] are bound to be different. Even if the training sequence of the original DUNET is the same as [8], the result of [8] and that of the original DUNET are different because the training dataset used is different and even the test dataset is different. In this experiment, we compare the result from approximate DUNET with the one from the original DUNET measured in our experimental environment.

#### 5.3.2. Transferability of Approximate DUNET to a Different DNN Model

The crux of DUNET is producing negative noises, which are anti-adversarial perturbations added on the adversarial images [8]. In this respect, it is possible to transfer the capability of DUNET to other models. The original DUNET is trained using Inception-V3 as a guide model, and Table 5 is the result when the target model to defend is Inception-V3. Evaluating transferability means that the target model to defend is not Inception-V3 while DUNET is the original DUNET guided by Inception-V3 in training. For transferability check, ResNet-152 [21] is used as the target model for DUNET to defend, and Table 6 shows the result.

Just as in the result in Table 5, for the case of transferability check, convolution kernel tensors with Tucker decomposition make negligible differences; both white-box and black-box cases were less than 0.49%. In the test result with clean images, almost no performance drop is measured (less than 0.2%).

### 5.4. Execution Performance Evaluation

In this subsection, we evaluate the execution performance of eDenoizer qualitatively and quantitatively. The experiment was conducted in two main execution environments. The first environment is when only the target model (Inception-V3) to be defended and DUNET to defend this target model are executed. The second environment is the case in which several other DNN models, including the aforementioned two models, are performed together. Basically, we compare the case of applying our solution (eDenoizer) with the case of running DNN models generated by the PyTorch framework without our solution (PyTorch).

The proposed eDenoizer consists of two solution parts: ➀ applying approximate convolutional kernel tensors to reduce the computation of DUNET and ➁ a priority-based GPU scheduling framework capable of parallel processing multiple DNN models. Thus, both experimental environments described in the preceding paragraph are subdivided into two aspects. (1) We measure the overall execution performance of eDenoizer, which includes both solution parts. (2) Two solution parts are then measured separately. In other words, we identify how each part of the solution is independently reflected in the overall execution performance. In each experiment, more than 100 images were continuously inputted to each DNN model including DUNET and the execution time of inference per image was averaged. The bars shown in each graph are the result of adding the denoizing time of DUNET and the inference time of the target model to be defended.

#### 5.4.1. Running Only the Defense Target Model and DUNET

**Figure 8:**
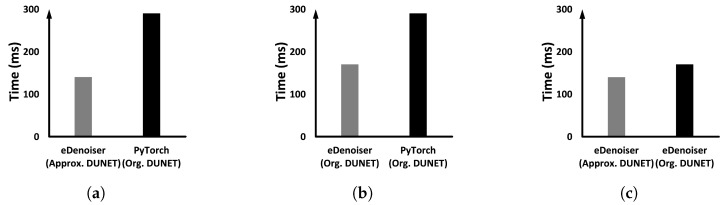
Execution time evaluation when only the target model to defend (Inception-V3) and DUNET are running: (**a**) comparing the overall performance depending on whether eDenoizer is applied or not, (**b**) identifying the impact of the proposed scheduling framework only and (**c**) identifying the effect of Tucker decomposition only.

Figure 8 demonstrates the results when only DUNET and the target model Inception-V3 are running together. Figure 8 compares the average target inference time $Inf_{avg}\left(F\right)$ which incorporates both the denoizing time of DUNET and the pure inference time of the defense target model, defined in Equation (2), under different conditions (a), (b), and (c). As shown in Figure 8a, when we apply both the approximate computing and the proposed scheduling framework together, the average inference time of the target model is reduced by 51.72%. Figure 8b is the case to only check the efficiency of the scheduling framework of eDenoizer separately, and the degree of reduction in the execution time reached up to 41.3%. Figure 8c displays only the effect of Tucker decomposition itself, and Tucker decomposition for kernel tensors accounts for about 17% of the total savings.

#### 5.4.2. Running Multiple DNN Models Together

**Figure 9:**
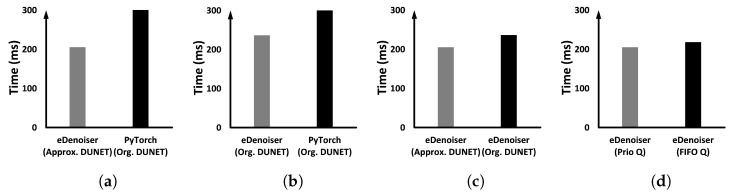
Performance interference evaluation when four DNN models in addition to the models used in Figure 8 are running together. We gave *H* priority to DUNET and the defense target model, while other co-runners have *L* priority: (**a**) comparing the overall performance depending on whether eDenoizer is applied or not, (**b**) identifying the impact of the proposed scheduling framework only, (**c**) identifying the effect of Tucker decomposition only and (**d**) performance comparison depending on the property of the job queue.

In addition to the DNN model used in the previous Section 5.4.1, several other DNN models were executed on the target device together to represent experimental scenarios closer to the actual situation. As for co-runners, we added ResNet-152 ×1 [21], RegNet ×1 [38], ResNext ×1 [39] and WideResNet ×1 [40]. We gave high priority to DUNET and Inception-V3 defended by DUNET, and low priority to the remaining four additional models. Through the experiment, we check whether the priority given through the CPU-side OS is reflected in the execution progress over the GPU. Figure 9 shows $Inf_{avg}\left(F\right)$ under different conditions (a), (b), (c), and (d), as the cases in Figure 8.

When we apply both the solution parts, as shown in Figure 9a, the average inference time of the defense target model is reduced by 48.36% compared to before applying eDenoizer. This clearly shows that eDenoizer suppresses performance interference from the less significant DNN models running together if DUNET and the defense target model have high priorities. This performance improvement of DUNET and the defense target model compared to before applying eDenoizer is primarily due to the effect that the priorities set on the host side were efficiently reflected on the GPU execution order and the job-based parallel processing capability of the proposed scheduling framework introduced into eDnoiser.

To check the effectiveness of the scheduling framework itself separately under the environment of multi-DNN running, we removed the Tucker decomposition from eDenoizer, and then compared the result with when we did not apply our solution. Figure 9b shows the result of it, and the obtained reduction is 40.55%. Next, we verify the Tucker decomposition effect in a multi-DNN execution environment. Using the scheduling framework of eDenoizer, we compare before and after Tucker decomposition is applied. In Figure 9c, we can see that there is a decrease in the execution time of about 13.13%. As the final experiment in this subsection, we compare the performance difference between a job queue as a FIFO queue and a priority queue. In this experimental step, the priorities provided by the CUDA stream are made the same, and only the results are measured when the property of the job queue is changed. Figure 9d shows the result, and applying the priority queue represents a performance advantage of about 6%.

#### 5.4.3. Applying Tucker Decomposition to the Defense Target Model

In the previous experiments, Tucker decomposition is applied only to DUNET and its computational scale is reduced. In contrast, this experiment analyzes the effect of applying Tucker decomposition to the defense target model in the same way. To do this, Inception-V3, ResNet-152, VGG-16 [22], and RegNet were first selected as defense target models, and after applying Tucker decomposition offline, each of the models is combined with DUNET to measure the inference execution time for each case. In this experiment, we basically use the scheduling framework of eDenoizer and measured the difference between using and not using Tucker decomposition for the defense target model.

**Figure 10:**
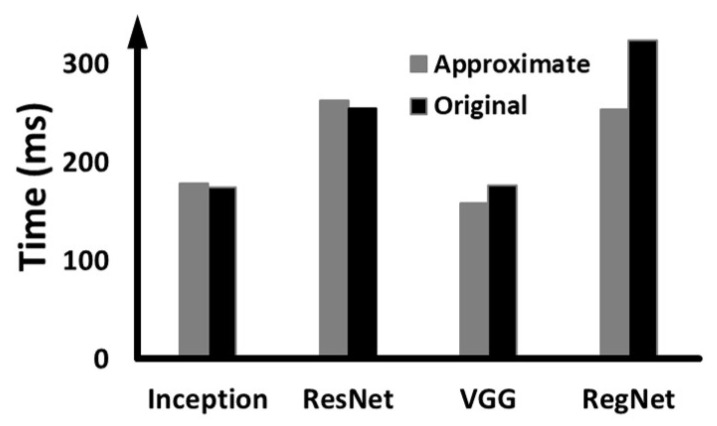
Execution time profiles when Tucker decomposition is applied to both the defense target model and DUNET.

Figure 10 shows the results, and ‘Approximate’ denotes the result of applying Tucker decomposition to the defense target models and ‘Original’ is for the case not applied. In the case of Inception-V3 and ResNet-152, the performance deteriorates when Tucker decomposition is applied. The inception module comprising Inception-V3 and the bottleneck building block comprising ResNet-152 are built up with 1 × 1 and 3 × 3 kernel tensors for dimension reduction. This is the same form as the core tensor and the factor matrixes shown in Figure 3. The 3 × 3 convolution kernel tensors in the inception module and the bottleneck building block already have a small enough number of output channels. Thus, applying Tucker decomposition to these 3 × 3 kernel tensors rather increases the overall computational scale as only factor matrixes (1 × 1 kernel tensors) are newly created and added. As a result, the inference time is delayed as shown in Figure 10. In contrast, when using VGG-16 and RegNet, which have relatively different structures, as the defense target models, there is a performance improvement of up to 21.6%.

**Figure 11:**
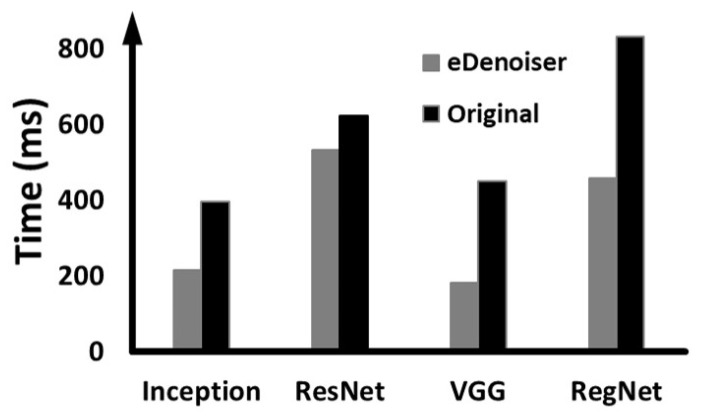
Performance interference evaluation: Tucker decomposition is selectively applied to the defense target models under the same experimental setup as Figure 9.

The following experiments examine the performance when several DNN models with different priorities are executed over one shared embedded GPU. The DNN models executed together and the priority assignment method are subject to the same conditions as in Section 5.4.2. Taking hints from the result of Figure 10, Tucker decomposition is applied only when VGG-16 and RegNet are used as the defense target model, and not for Inception-V3 and ResNet-152. Figure 11 shows the overall experimental results. As can be seen from the figure, when VGG-16 is the defense target model, eDenoizer is able to reduce the time required for adversarial defense and inference by 59.86%. In conclusion, under more real-world conditions where several DNN models run together, the proposed eDenoizer effectively guarantees performance isolation in terms of inference time accompanied by adversarial defense.

### 5.5. Memory Footprint Reduction

Experiments so far examined the inference accuracy drop and the improved execution speed through the proposed eDenoizer. In this subsection, we take a look at the utility in terms of the system metric as the last quantitative verification.

Tucker decomposition reduces computation. That is, the execution time is accelerated by reducing the burden of the GPU performing the DNN operations. In addition, Tucker decomposition reduces the number of parameters of DNN models. In this experiment, we measure memory footprint reduction. It is worth noting that GPUs used in IoT edge devices such as Jetson AGX Xavier and Jetson TX2 do not have a separate dedicated memory and share DRAM, a system memory, with CPUs. Therefore, memory saving is very significant in IoT edge devices.

Table 7 shows the memory reduction result where Approximate means the result of the models with Tucker decomposition and Original is for the unmodified DNN models. First of all, in the case of DUNET, there is an effect of reducing memory usage by about 18% when applying Tucker decomposition. Among the defense target models, Inception-V3 showed the largest decrease of 43%. However, as shown in Figure 10, in Inception-V3 and ResNet-152 cases, applying Tucker decomposition increases the execution time slightly. Therefore, considering the reduction in execution time and memory footprint at the same time, it can be said that it has 9% effect on VGG-16 and 27.2% effect on RegNet.

## 6. Related Work

Our work proposes a solution that can handle adversarial defenses inside IoT edge devices with minimal latency. Research works related to this can be organized into two areas: the field of structural modification of deep learning models for resource constraint devices and the improvement of the computational efficiency to expedite the inference phase of deep learning models.

### 6.1. Lightweight Deep Learning Model over the Structural Change

Studies in this category discussed model compression techniques maintaining the minimized accuracy compromise. The main idea is cutting out unimportant layers, filters, and channels constituting DNN models. By doing so, the simplified model allows for less power consumption and reduced computational amounts compared to the original model. Then, the compressed DNN models are trained with the datasets used in the training steps of the original models (fine-tuning) to compensate for structural deterioration.

MobileNets [41] introduced depth-wise separable convolution, which allows each input channel to have a filter assigned to it. The existing 3 × 3 convolution was changed to one 3 × 3 depth-wise convolution and one 1 × 1 point-wise convolution. Through this, the amount of computation and the number of parameters were reduced. To trade-off between the latency performance and the accuracy result, [41] also provides two global hyper-parameters.

For lightweight face recognition, [42] applied knowledge distillation. This technique enhances the interpretation ability of the network. To relieve the generalization gap between teacher and student models, [42] proposes recursive knowledge distillation. To secure architectural flexibility, the authors of [42] used knowledge distillation instead of quantization or pruning.

DeepMon [43] focuses on on-device continuous vision applications without network delay and privacy concerns. DeepMon tried to achieve the energy efficiency and the minimum delay in the inference step on the target device, Galaxy S7. In particular, DeepMon suggested an optimization technique that can effectively offload the convolution operation, generally called upon as performance bottleneck, to the mobile GPU.

Deep Compression [44] proposed a deep learning model compression technique by reducing the required number of bits represented in weight matrixes. [44] basically consists of three steps: (1) Pruning the model by removing the useless connection while maintaining only the connection that contains a lot of information. (2) Putting weights with similar values in the same bin so that they have the same weight, and weight sharing is possible by saving only the index of the bin. (3) Huffman coding expresses the most frequent weight value in a small bit.

EIE [45], which further developed the result of [44], proposed an hardware architecture especially designed for the pruned deep learning model. Weight matrixes are converted to have 4∼25% sparsity, and weight values are limited to several types of values. By k-means clustering, relatively close weight values are expressed as one value as in [44]. Basically, EIE consists of a CCU (central control unit) and multiple PEs (processing elements). EIE calculates only non-zero activation values and enables parallel operations via PEs, providing both the energy efficiency and the inference speed-up at the same time.

Ref. [46] proposed a linear integer quantization technique. Ref. [46] performs the inference step only using integer arithmetic instead of floating-point operations since the integer arithmetic needs a smaller number of bits. Ref. [46] addressed that the prior quantization techniques lacks in terms of on-device consideration, i.e., prior research works only assumed corresponding accelerators. Ref. [46] provides a quantized inference framework for integer-arithmetic-only cores embedded in mobile devices. Ref. [46] also provides quantization-aware training through fake quantization technique.

Ref. [47] explored the relationship between the quantization technique and adversarial attacks. Ref. [47] proposed and used ANS (adversarial noise sensitivity) to identify the optimal bit-width per each layer required to defense adversarial attacks. If a layer has high ANS value, it is quantized to have smaller bit-width and in the opposite case, low ANS valued layers are maintained at larger bit-width.

Our proposed approach maintains the same approach in terms of keeping the computational scale of the deep learning model small, but different in terms of using tensor-decomposition scheme. Further, our approach differs from the aforementioned approaches in that we propose a methodology that can efficiently control the computation unit of the deep learning model at the middleware level.

### 6.2. Enhancing the Computational Efficiency

#### 6.2.1. Hardware Acceleration

Research works in this class tried to offer particular computing units that can perform inference tasks much more rapidly than general purpose cores such as CPUs and GPUs. The NVIDIA Deep Learning Accelerator (NVDLA) project provides an open architecture to satisfy the computational demands of hardware accelerated inference [48]. Since the architecture of NVDLA is based on a modular design, NVDLA can give developers flexibility and easy integration methodology. NVDLA supports five basic hardware components which are independently configurable: convolution core, single data processor, planar data processor, channel data processor, and dedicated memory and data reshape engines. Convolution core is for highly optimized execution of convolutional layers, single/planar/channel data processor is for activation/pooling/normalization function, and dedicated memory and data reshape engine is for tensor reshape and memory-to-memory data transfer. This component-level acceleration technique allows IoT edge designers to achieve high-performance execution of deep learning models.

To provide acceleration especially in matrix operation, NVIDIA also supports Tensor cores. Tensor core is a GPU core that performs 4 × 4 matrix operations. While CUDA core performs one fp32 on one GPU clock, Tensor core performs a matrix multiplex-accumulate operation that multiplies two 4 × 4 fp16 matrixes and adds the result to the 4 × 4 fp32 matrix on one single GPU clock [49].

Processors that accelerate neural network-specific operations are usually referred to as NPUs (neural processing units), and DianNao series is a typical example. DianNao [50] is the first architecture that deals with the deep learning accelerator, and is composed of NFU (neural functional unit), buffer, and CP (control processor). NFU, operating in 16 bit fixed point, takes over convolution, pooling, and classification layers. Buffer is for input/output neurons and synaptic weight values. CP is a CPU-like component and is responsible for the overall control. DaDianNao [51] is extended DianNao to use eDRAM (embedded DRAM). By using eDRAM, latency is reduced and high bandwidth can be provided. In ShiDianNao [52], through a network specialized for data transfer, adjacent PEs (processing elements) may exchange data with each other.

Eyeriss [53] is a CNN accelerator, which considers in-depth minimizing the energy cost incurred by data movement while maintaining high performance. Eyeriss proposed a dataflow-driven processing mechanism called RS (row stationary). Using RS dataflow technology, Eyeriss can reduce the number of DRAM access as much as possible and allows data reuse and partial sum accumulation through memory hierarchy.

To support the especially required computation in defending against adversarial attacks, DNNGuard [54], structured with RISC-V [55] and NVDLA, is proposed. DNNGuard, basically a hardware accelerator, can execute simultaneously the original target deep learning models and the adversarial detection network such as NIC [56]. DNNGuard incorporated the CPU core and deep learning accelerator hardware into one single chip. Thus, latency-optimized data transfer can be guaranteed. DNNGuard also proposed an extended instruction set to configure dynamically the PEs (processing elements) and the internal memory, and to enable efficient data interaction between the currently running deep learning model and the adversarial detection network. DNNGuard [54] has the same side as our approach in terms of acceleration of adversarial defense, but our approach does not involve additional hardware.

#### 6.2.2. Software Techniques for Efficient Use of Existing Computing Units

DeepSense [57] is a OpenCL-based framework efficiently designed for CNN operations on a mobile GPU. To cope with various types of representation of deep learning models, a model converter translates the models into a predefined format. Then, a model loader loads the converted models into the CPU and GPU memory. A inference scheduler controls the multiple submitted DNN kernels, and an executor takes care of the execution pipeline over the CPU and the GPU. DeepMon [43] extended DeepSense by providing more optimization techniques and comprehensive evaluation.

Neurosurgeon [58] suggests a mechanism to partition the workload of deep learning inference properly. At development stage, Neurosurgeon runs the target DNN model once per each mobile device and cloud server to produce layer time execution prediction models for different DNN layers. Then, at runtime stage, Neurosurgeon uses the prediction models generated in the development stage to choose the best partition point. Finally, partitioned execution of the target DNN model is performed using both the mobile device and the cloud server.

Ref. [59] significantly improves the speed of calculation of acoustic parameters in Zwicker’s psycho-acoustic nuisance model on IoT devices such as Raspberry Pi [60]. It is very challenging to accurately calculate soundscape profiling in real time within a traditional WASN (wireless acoustic sensor networks) environment. The authors of [59] used an end-to-end CNN-based solution to allow calculation of four PA (psycho-acoustic annoyance) parameters 250 times faster than conventional algorithms.

$S^{3}$DNN [61] proposed the two-stage system-level optimization technique. In the first stage, called front-end, data of DNN workloads are fused by selectively fusing multiple input video frames. At this stage, $S^{3}$DNN checks whether the data fusion satisfies the real-time constraint and guarantees the maximum throughput. During the next stage, actual computation is carried out. Here, $S^{3}$DNN tries to execute the fused video frames optimally through supervised streaming and GPU kernel scheduling enabled by the CUDA stream technology of NVIDIA.

To ameliorate the latency performance of on-device DNN-assisted applications, μLayer [62] and ODMDEF [27] provide seamless DNN-computation across heterogeneous cores which are operated in different ISA (instruction set architecture). The motivation of these two studies is that the CPU core and the GPU core in the same embedded system generally represent comparable computational throughput. μLayer is a runtime, and make the CPU and the GPU execute the disjoint sets of the channels of a DNN layer. Based on the fact that native hardware supports of both heterogeneous cores are different, μLayer applied 8-bit integer to the CPU and 16-bit floating point to the GPU. μLayer also concurrently executes parallelizable branches in a DNN layer (e.g., inception modules of Inception-V3 [20]) by allocating them to both heterogeneous cores. ODMDEF [27] is a CPU-GPU co-scheduling framework. Since DNN workloads are requested irregularly, ODMDEF performs core type selection for an instant DNN workload in a way of dynamic decision. This decision takes into account the degrees of current use of the CPU and the GPU, and the expected execution times of the workload on both core types. ODMDEF also minimizes the data-transfer overhead incurred in data-synchronization steps between the two core types, and as $S^{3}$DNN [61], exploits the CUDA stream technology for the maximum parallelization effect inside the GPU.

When it comes to effectively using existing hardware to increase the DNN computational efficiency, the above listed studies in this category can be said to be similar to our approach. However, our work is a hybrid form of work that modifies the computational structure of DNNs and applies priority-aware multi-DNN scheduling solutions. Furthermore, it differs in that it is a study that can solve the problem in time-constrained adversarial defense with a software-only solution.

## 7. Conclusions

Adversarial attacks can lead to catastrophic results, messing up the classification behavior of IoT edge devices. In adversarial defense to prevent this, immediate denoizing must be accompanied in the overall inference process because the inference phase of the defense target model proceeds after removing adversarial noises. In an IoT edge device environment, multiple DNN models share and use an embedded GPU, which handles DNN kernels with a FIFO order and, furthermore, is a non-preemptive device, so time-constrained adversarial defense is highly challenging.

To solve this problem, this study proposed eDenoizer. It first efficiently cuts down the computation by decomposing the convolutional kernel tensors of the denoizer by 25.41%. The scheduling framework of eDenoizer embraces a C++-based multi-threading technique in which each DNN operation can be assigned to one of the most appropriate CUDA streams, which is not possible in pure Python execution environment. In addition, to minimize performance interference from low-priority DNN models performed together, the priority specified by the user can be reflected not only on the CPU side but also on the GPU computation order. Through these proposed techniques, the denoizer and the defense target model are efficiently parallelized and also preferentially executed in the GPU. After measuring and analyzing using various experimental methods, the reduction of classification accuracy was negligible (less than 1.78%), and obtained speed-up in the inference time was up to 51.72%.

In this study, the size of the scheduled job was manually determined offline after the user identified the characteristics of each DNN. In future studies, we plan to add the feature that each DNN characteristic is identified by the system itself and then automatically finds the optimal scheduling unit.

## Figures and Tables

**Table 1:** Execution time taken for each of the DNN models when processing a single input image.

	Inception-V3	ResNet-152	VGG-16	DUNET
**Jetson AGX Xavier**	86.3 ms	157.7 ms	50.4 ms	160.7 ms
**Jetson TX2**	103.9 ms	198.6 ms	76.8 ms	246.4 ms

**Table 2:** Specification of the Jetson AGX Xavier platform [9,27].

	Classification	Description
HW	CPU	8-core ARM v8.2 Carmel 64-bit CPU, 8 MB L2, 4 MB L3 cache
	GPU	512-core Volta GPU with Tensor cores
	Memory	32 GB 256-Bit LPDDR4x, 137 GB/s
	Storage	32 GB eMMC 5.1
SW	Kernel Ver.	Linux 4.9.140
	SW Package	JetPack 4.2
	CUDA Ver.	CUDA v10.0.166
	Denoiser	DUNET in HGD [8]

**Table 3:** Adversarial images for training and validation [8].

	Attack Method	Attacked Model
Training Set and Validation Set	FGSM	IncV3
	FGSM	IncResV2
	FGSM	Res
	FGSM	IncV3/IncResV2/Res
	IFGSM2	IncV3/IncResV2/Res
	IFGSM4	IncV3/IncResV2/Res
	IFGSM8	IncV3/IncResV2/Res

**Table 4:** Adversarial images for testing [8].

	Attack Method	Attacked Model
**White-box-test-set**	FGSM	IncV3
	IFGSM4	IncV3/IncResV2/Res
**Black-box-test-set**	FGSM	Inception-V4
	IFGSM4	Inception-V4

**Table 5:** Comparing the classification accuracy.

	Result in [8]	Org. DUNET	Approx. DUNET
**Clean-image-test-set**	76.2%	76.53%	76.38%
**White-box-test-set**	75.2%	72.37%	70.59%
**Black-box-test-set**	75.1%	74.86%	74.45%

**Table 6:** Transferability to different model (ResNet-152).

	Result in [8]	Orginal DUNET	Approximate DUNET
**Clean-image-test-set**	77.4%	73.7%	73.5%
**White-box-test-set**	75.8%	71.35%	70.86%
**Black-box-test-set**	76.1%	72.07%	71.58%

**Table 7:** Memory footprint reduction by Tucker decomposition.

	Inception-V3	ResNet-152	VGG-16	RegNet	DUNET
**Original**	105 MB	231 MB	528 MB	555 MB	43 MB
**Approximate**	59 MB	144 MB	481 MB	404 MB	35 MB

## Data Availability

Publicly available datasets were analyzed in this study. The data can be found in this links: https://www.image-net.org/ (accessed on 15 March 2022).
